# Advances in natural-product-based nanomaterials for treatment of acute lung injury

**DOI:** 10.3389/fbioe.2025.1663961

**Published:** 2025-09-01

**Authors:** Jiajing Yang, Yenna Hsu, Rui Liu, Dan Chen, Zhihang Zhou, Jingshan Zou, Peizheng Xiong, Li Zhou

**Affiliations:** ^1^ Chengdu University of Traditional Chinese Medicine, Chengdu, China; ^2^ Department of Otorhinolaryngology, Hospital of Chengdu University of Traditional Chinese Medicine, Chengdu, China

**Keywords:** acute lung injury, anti-inflammatory, nanomaterials, natural products, review

## Abstract

Acute lung injury (ALI) is a critical condition characterized by rapid-onset lung inflammation, often resulting in respiratory distress. Current treatments are mainly based on glucocorticoids, but side effects and variable efficacy limit their effectiveness. This has prompted research into novel treatments, focusing on natural-product-based nanomaterials (NP-NMs), which offer a promising alternative. NP-NMs, synthesized from biological sources such as plants and microorganisms, have shown potential in therapy of ALI by enhancing drug delivery, reducing systemic side effects, and modulating inflammation. This review summarizes the latest research on NP-NMs, highlights their advantages in terms of biocompatibility, targeted delivery, and overcoming biologic barriers, and explores the challenges of developing NP-NMs in terms of standardized synthesis methods, comprehensive toxicological evaluation, and optimization for clinical translation. The significance of this review is to provide ideas for the development of more effective treatments for ALI, supporting further investigation into their clinical applicability.

## 1 Introduction

Acute lung injury (ALI) is a respiratory disease characterized by severe hypoxemia and diffuse alveolar damage (He et al., 2021). The primary clinical symptoms include shortness of breath, respiratory distress, and hypoxemia. A chest X-ray reveals diffuse infiltrative shadows in both lungs. ALI can deteriorate into a severe condition, known as acute respiratory distress syndrome (ARDS) (Bein et al., 2018; Cheng et al., 2021; Sasidharan et al., 2019). Globally, ARDS has been reported to account for 10.4% of all admissions to intensive care units and 23.4% of cases requiring mechanical ventilation, and it has a high in-hospital mortality rate of 35%. It is therefore a serious threat to public health (Bellani et al., 2016). Currently, the most effective treatments for ALI involve medical interventions that target the reduction of pulmonary and systemic inflammation, combined with supportive care in the form of mechanical ventilation (Fan et al., 2018; Zhang J. et al., 2024). Nevertheless, these therapeutic approaches are subject to several constraints, including strategies of low tidal volume ventilation, which may result in carbon dioxide retention or inadequate blood oxygenation, the absence of specific medications, and the lack of safer or more efficacious treatment options.

The limitations of the abovementioned clinical therapies have necessitated the development of more promising drugs for treating ALI. In recent years, an increasing number of natural products and their derivatives have been shown to have therapeutic potential for treating ALI. Increasingly compelling evidence suggests that natural products, derived from a diverse array of sources such as plants, animals, microorganisms, and marine life, which have been subjected to natural selection and evolution within their unique environments, exhibit significant bioactivity and adaptability (Chopra and Dhingra, 2021; Luo et al., 2024). They play an important role in multiple pathological stages of ALI due to their structural diversity and multiple biological activities, such as anti-inflammatory, antioxidant, and immune regulation. In contrast, most compounds may have limited therapeutic effects due to their single target design. In addition, natural products often have low toxicity and good metabolism in the body, making them safer for the treatment of ALI. However, owing to their unique physicochemical properties, these products have shortcomings such as low solubility and limited bioavailability. Therefore, it is necessary to identify appropriate drug delivery methods to improve their utilization rates and thereby achieve the desired therapeutic effects (Xu et al., 2015; Cao et al., 2020; Cao et al., 2022).

Nanotechnology involves methods of manipulating and controlling matter on the nanoscale (Cao et al., 2022; de Alcantara Lemos et al., 2021). Research on nanomaterials is rapidly developing, and these materials are widely applied in areas including medicine, electronics, and materials science. Many studies have shown that nanomaterials greatly facilitate the treatment of respiratory diseases in ways such as improving drug solubility, increasing drug bioavailability, and achieving targeted drug delivery (Peng et al., 2024). Consequently, by integrating nanomaterials with natural substances may holds the potential to enhance the bioavailability of medications.

**FIGURE 1:**
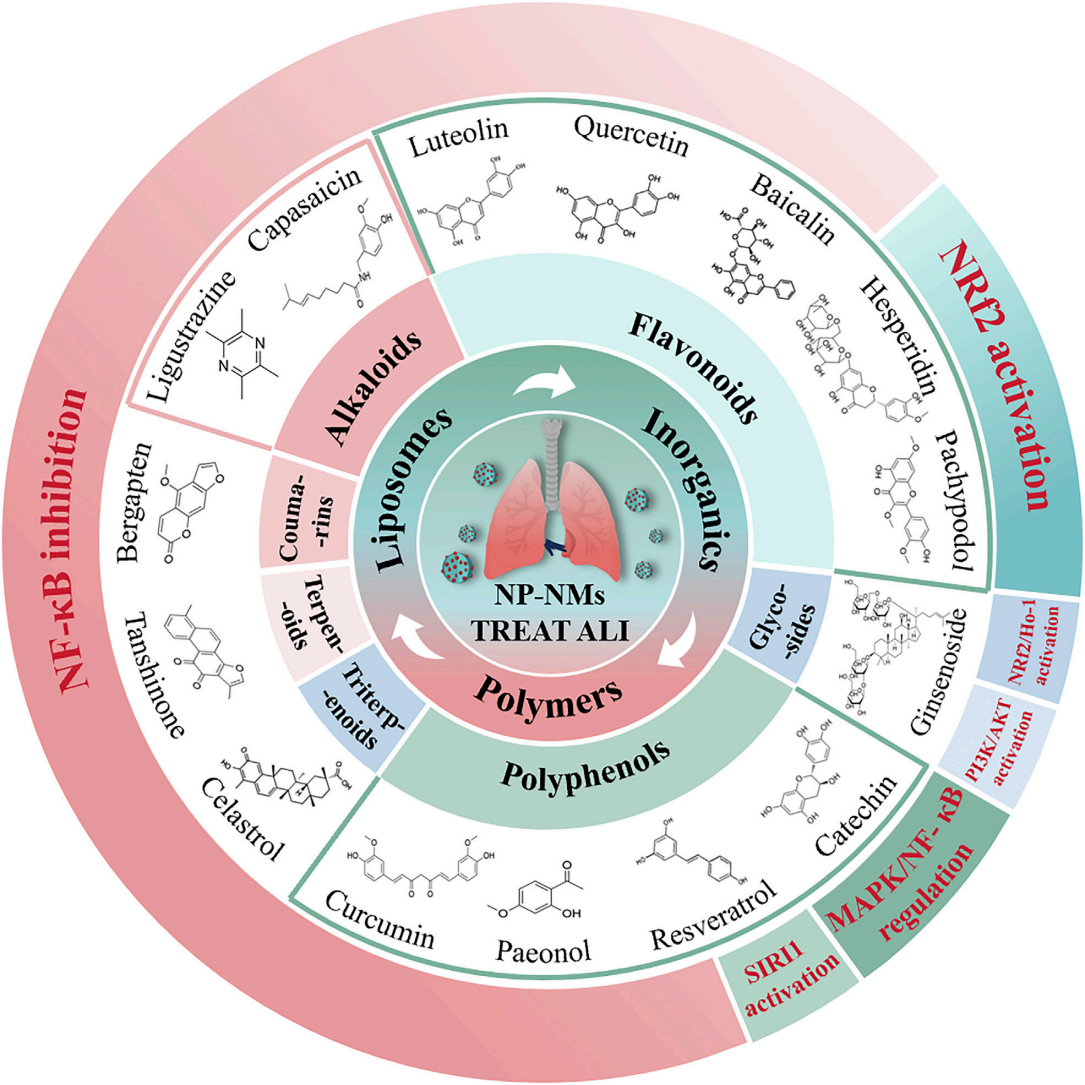
The illustration of the potential therapeutic role of natural product-based nanomedicines for ALI is presented. These are mainly categorized into alkaloids, flavonoids, polyphenols, triterpenoids, glycosides, terpenoids, and coumarins.

In this review, we summarize recent progress in the use of natural product-based nanomaterials (NP-NMs) for the treatment of ALI, and we analyze the underlying principles of their therapeutic action. Additionally, we examine recent studies on the use of nanocarriers for treating ALI, drawing upon an original overview, and provide a concise summary of their mechanisms in addressing ALI. Lastly, we discuss the challenges associated with the development of NP-NMs, aiming to facilitate their advancement toward becoming a viable therapeutic option for ALI in the future (Figure 1).

## 2 Pathogenesis and current treatment of ALI

It is widely recognized that uncontrolled inflammation in the lungs or throughout the body is the primary cause of the pathogenesis of ALI and ARDS (Dechert et al.). Inflammation caused by infectious agents, poisons, or trauma is thus an important factor in the development of ALI. Macrophages represent the first line of defense and play a major role in inflammation (Aggarwal et al., 2014; Bian et al., 2021a). When exposed to inflammatory stimuli, resting macrophages (M0) become activated and differentiate into either a proinflammatory phenotype (M1) or an anti-inflammatory phenotype (M2). This causes the release of proinflammatory substances such as tumor necrosis factor (TNF)-α, interleukin (IL)-1β, and IL-6 (Bian et al., 2021a; Huang et al., 2018; Sica and Mantovani, 2012).

Moreover, neutrophils, epithelial cells, and endothelial cells are also involved in the inflammatory response (Silva and Rocco, 2017). Under the influence of chemotaxis, neutrophils are the first cells to disrupt the epithelial–endothelial barrier. They release cytotoxic mediators such as reactive oxygen species (ROS) in this process, which contributes to the inflammatory response. Nanomedicines have the potential to alleviate lung injury by inhibiting the production of proinflammatory cytokines and reducing neutrophil aggregation (Potey et al., 2019). Furthermore, lung injury can result in the dissociation of intercellular junctions within the lung epithelium, trigger cell death, and induce apoptosis of endothelial cells, which further leads to impaired lung function. Nanomedicines may help to preserve the integrity of the epithelial–endothelial barrier by reducing cell death (Lucas et al., 2009; Jiang et al., 2020; Short et al., 2016; Lin et al., 2013). Neutrophils, alveolar macrophages, endothelial cells, epithelial cells, etc., are activated in lung injury, and their release of large amounts of ROS leads to the development of oxidative stress. The release of ROS creates a feedback loop that further activates inflammatory cells and perpetuates the cytokine storm (Potey et al., 2019; Zhang et al., 2020). Numerous inflammatory signaling pathways, such as the nuclear factor kappa B (NF-κB), mitogen-activated protein kinase (MAPK), toll-like receptor (TLR), and Janus kinase (JAK)/signal transducer and activator of transcription (STAT) pathways, are activated when ROS are produced (Yeung et al., 2018). Therefore, nanomedicines can be used as therapeutic agents for ALI and reduce oxidative damage to lung tissue by inhibiting various inflammatory pathways and suppressing ROS-induced oxidative stress.

In 2019, the outbreak of coronavirus disease 2019 (COVID-19) emerged as a major global health crisis, significantly impacting human health and social development. COVID-19 is caused by infection with the enveloped, positive-sense, single-stranded RNA virus SARS-CoV-2. SARS-CoV-2 infection exhibits a broad tendency across various tissues; however, most severe SARS-CoV-2 infections are typically associated with extensive lung damage, which can lead to pneumonia, acute respiratory failure, and death. Research has found that natural products exert pre- and post-infection inhibition, multi-stage inhibition, immune regulation, and combined targeted therapy effects, which have a positive effect on lung damage caused by COVID-19 (Low et al., 2023; Singh and Sodhi, 2023).

In addition, pulmonary endothelial cells and vascular endothelial growth factor (VEGF) have important roles in ALI. VEGF induces lung endothelial cells to synthesize and release prostacyclin, nitric oxide (NO), and inflammatory mediators such as TNF-α, IL-1β, and IL-8 (Chicione et al., 2011; Gill et al., 2015). In addition, VEGF can enhance angiogenesis and increase microvascular permeability by binding to vascular endothelial growth factor receptor and can thereby alleviate structural lung injury. This provides a rationale for the possible utilization of nanomedicine-delivered endothelial growth factor for the treatment of ALI (Cahill and Redmond, 2016).

**FIGURE 2:**
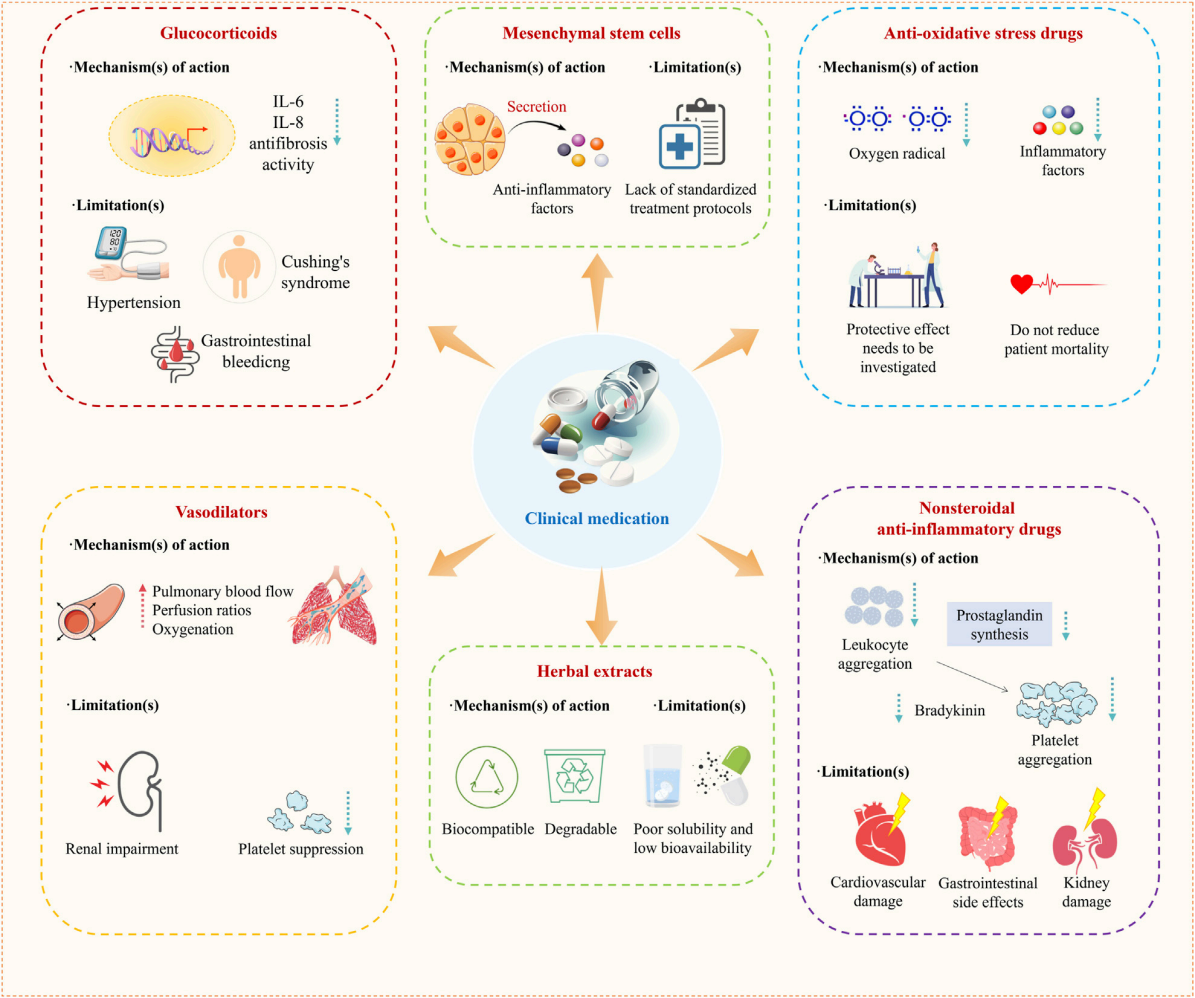
Mechanisms and limitations of current clinical drugs.

Treatments of ALI are mainly categorized into nonpharmacological mechanical ventilation and pharmacological treatments (Zhang J. et al., 2024; Guo et al., 2016). For the purpose of respiratory support and improving the patient’s oxygenation levels, it is appropriate to adopt a strategy of lung protective ventilation using a low tidal volume (less than 6 mL/kg predicted body weight) combined with a limited inspiratory plateau pressure, thus preventing lung hyperinflation (Zhang J. et al., 2024). In drug therapy, anti-inflammatory drugs are commonly used in clinical practice. Among them, glucocorticoids are the most widely used (Figure 2). In addition, nonsteroidal anti-inflammatory drugs (NSAIDs) (Bian et al., 2021a; Matthay et al., 2019; Hamid et al., 2017), N-acetylcysteine (NAC) (Hecker, 2018; Sarma and Ward, 2011), Vasodilators are also commonly used drugs in the clinical treatment of ALI (Searcy et al., 2015) (Figure 2). In recent years, mesenchymal stem cells, herbal extracts, and intestinal flora have also emerged as new options for treating ALI (Figure 2) (Table 1) (Qu et al., 2024; Fernández-Francos et al., 2021; Liu et al., 2022).

**TABLE 1:** Mechanisms and limitations of current clinical drug.

Medication	Mechanism(s) of action	Limitations(s)	Ref.
Glucocorticoids	Downregulation of transcription of genes encoding proinflammatory cytokines such as interleukin (IL)-1 and IL-8 and antifibrosis activity	Clinical benefits are unclear, and there are various side effects such as hypertension, Cushing’s syndrome, and gastrointestinal bleeding	Meduri et al. (2020), Rhen and Cidlowski (2005)
Anti-oxidative stress drugs	Scavenging oxygen-containing free radicals and reducing levels of inflammatory factors	Do not reduce patient mortality, and the exact protective effect needs to be investigated	Zhang et al. (2017)
Nonsteroidal anti-inflammatory drugs	Inhibition of platelet aggregation by inhibiting leukocyte aggregation and prostaglandin synthesis and reduction of bradykinin formation, thereby exerting anti-inflammatory effects	Common side effects include gastrointestinal side effects, kidney damage, and cardiovascular damage	Southworth et al. (2015)
Vasodilators	Improving pulmonary blood flow, perfusion ratios, and oxygenation	Do not reduce mortality, and common side effects include platelet suppression and renal impairment	Gebistorf et al. (2016)
Mesenchymal stem cells	Secretion of anti-inflammatory factors, which directly suppresses the inflammatory response	Lack of standardized treatment protocols	Adir et al. (2008)
Herbal extracts	Biocompatible and degradable	Poor solubility and low bioavailability	Wang et al. (2024)

## 3 Research on the mechanisms of nanomaterials used for the treatment of ALI

**FIGURE 3:**
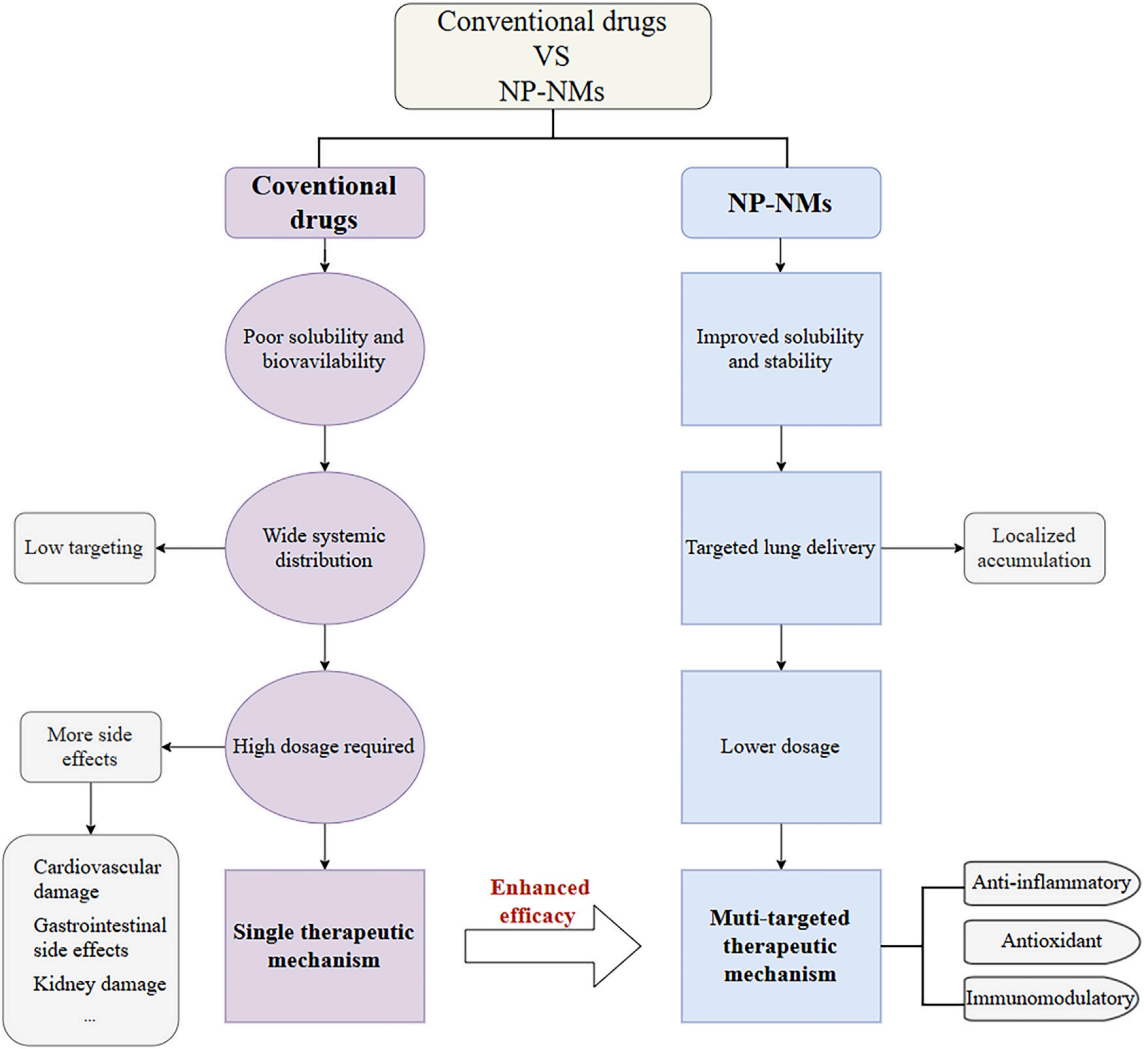
Flowchart comparing conventional drugs and NP-NMs.

The pathogenesis of ALI is complex and multifactorial, involving an imbalance in the inflammatory response, dysregulation of endothelial cell function, and the effects of vasoactive substances. Investigation of the mechanisms of nanomedicines used in the treatment of ALI is beneficial for more effectively exploring their potential. Compared to conventional drugs, natural product-based nanomaterials (NP-NMs) exhibit significantly optimized pharmacological properties. Conventional drugs often suffer from poor solubility and bioavailability, leading to wide systemic distribution and low targeting efficiency. As a result, high doses are required to achieve effective therapeutic outcomes, which increases the burden on the cardiovascular, gastrointestinal, and renal systems. Additionally, traditional drugs typically act through a single therapeutic mechanism, limiting their clinical efficacy. In contrast, NP-NMs enhance drug solubility and stability, enabling targeted delivery to the lungs and promoting local drug accumulation. This targeted delivery allows for reduced doses while maintaining therapeutic efficacy, thereby minimizing systemic side effects. NP-NMs often exhibit multi-targeted therapeutic effects, including anti-inflammatory, antioxidant, and immunomodulatory activities, which collectively contribute to their superior efficacy. By summarizing a large number of studies, it has been found that nanomaterials mainly treat ALI via the following mechanisms (Figure 3).

### 3.1 Anti-inflammatory mechanisms

**FIGURE 4:**
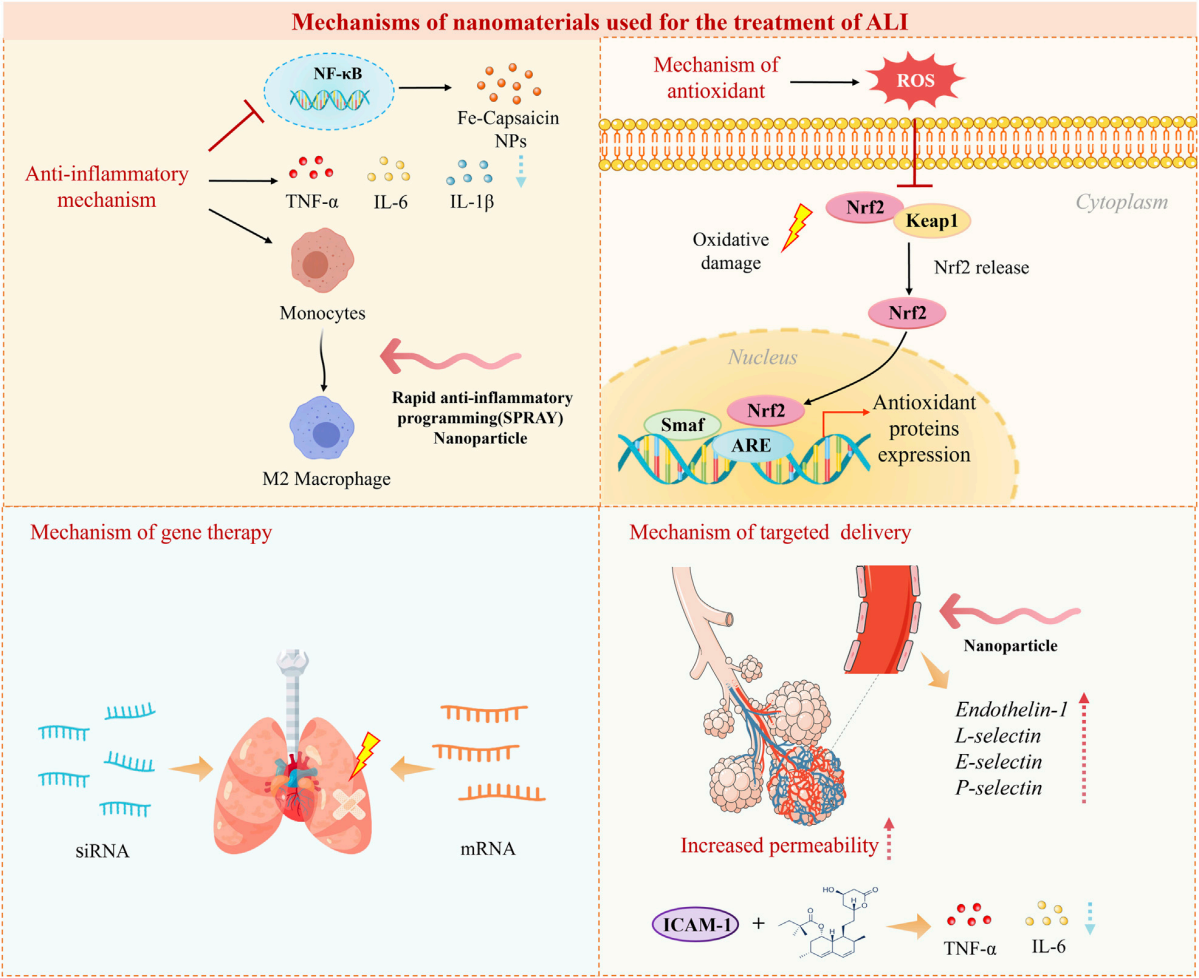
Mechanisms of nanomaterials used for the treatment of ALI.

The cellular inflammatory storm is one of the main causes of severe inflammation in ALI and leads to the release of various inflammatory factors such as TNF-α, IL-6, and IL-1β (Figure 4). A promising approach is to exert anti-inflammatory effects by inhibiting the secretion of pro-inflammatory cytokines. For example, ROS-responsive polythione nanoparticles (NPs) loaded with dexamethasone (DEX) have been shown to significantly reduce the levels of the pro-inflammatory factors IL-6, TNF-α, and IL-1β. This reduction exerts an inhibitory effect on inflammation and helps alleviate acute lung injury (Zhai et al., 2022). Nanomaterials can exert anti-inflammatory effects by modulating macrophage polarization (Figure 4). One research team constructed novel NPs comprising self-assembling peptides for rapid anti-inflammatory programming (SPRAY), which self-assemble to form NPs, by screening to identify the best SPRAY candidate, namely, the peptide BLKR (Chen D. et al., 2024). These NPs were experimentally found to specifically target alveolar macrophages to promote M2 polarization and inhibit M1-related signaling. Inhibition of signaling pathways is also one of the ways in which nanomaterials exert anti-inflammatory effects, with the NF-κB signaling pathway being a central regulator of the inflammatory response (Figure 4) (Jing et al., 2015). NF-κB is the main regulatory pathway of the inflammatory response. The expression of proinflammatory cytokines and chemokines depends on NF-κB, and the expression of these factors activates neutrophils, leading to increased alveolar capillary permeability, manifested as pulmonary edema and oxygenation disorders. When ALI occurs, NF-κB can be activated by multiple factors, and its sustained activation can lead to an inflammatory storm, thereby exacerbating ALI (Zhou et al., 2023). It has been found that iron-capsaicin nanozymes are able to alleviate sepsis-induced ALI via the NF-κB signaling pathway (Wang R. et al., 2024).

### 3.2 Antioxidant mechanisms of action

Oxidative stress plays an important role in the development of ALI due to endothelial damage and capillary leakage. Nanomaterials can mitigate lung damage resulting from excessive oxidative stress by increasing antioxidant capacity and reducing the production of oxygen-containing free radicals (Laforge et al., 2020). Strategies have been developed to modulate the oxidative microenvironment in ALI using ROS-responsive nanoparticles (NPs). For instance, Muhammad et al. prepared DEX-loaded polyurethane (PFTU@DEX) NPs by a modified emulsification method and investigated their effects by using an ROS-specific bioluminescent probe (L012) to assess ROS levels in different groups in a mouse model. They concluded that the PFTU@DEX NPs significantly reduced the local expression of ROS and thus alleviated ALI (Muhammad et al., 2022). In addition, some nanomaterials possess endogenous antioxidant mechanisms. The Nrf2/antioxidant response element (ARE) signaling pathway plays an important role in the activity of endogenous antioxidants, as Nrf2 induces the action of ARE genes to prevent the production of excessive ROS (Figure 4). Recent studies have shown that various metal-based NPs (e.g., Au, TiO_2_, and Ag NPs) can stimulate the Nrf2/ARE signaling pathway to achieve an antioxidant effect by increasing the levels of ROS and malondialdehyde (Xiong et al., 2022).

### 3.3 Mechanisms of gene therapy

Gene therapy is a new type of treatment that replaces abnormal genes with normal genes, introduces new genes, or edits existing genes to combat specific diseases (Verma and Somia, 1997). Nanomaterials can be used as carriers for targeted delivery of gene drugs, retarding their degradation, extending their half-life, and improving the efficiency of their action (Salameh et al., 2020; Xin et al., 2017; Miyazawa et al., 2017; Alex et al., 2013). The delivery of specific siRNAs is a new strategy with potential for the treatment of ALI in clinical practice. Nanomaterials can deliver specific siRNAs to inhibit the expression of inflammatory genes (Figure 4). Chen et al. constructed a selenium nanozyme therapeutic system (cationic water-soluble pillar arene [CWP]-Se@mannose [Man]) loaded with C-C chemokine receptor 2 (CCR2)-siRNA. This system exhibited electroneutrality and targeting properties for lung macrophages. It was observed that the mucus permeability of CWP-Se@Man was increased by a factor of about 15. CWP-Se@Man was able to effectively penetrate the mucus layer of the lungs to deliver CCR2-siRNA into macrophages. Moreover, in an inflammatory situation, the CWP-Se@Man nanotherapeutic system loaded with CCR2-siRNA could exert an inhibitory effect on chemotaxis and was able to scavenge ROS, which could alleviate ALI (Chen X. et al., 2024). LNPs have great potential for the delivery of mRNA, and studies of LNP-mRNA complexes for the treatment of ALI are becoming increasingly widespread. Research on the inhalation delivery of mRNA also shows great potential (Jiang et al., 2024). However, LNP-mRNA complexes have the disadvantage of a low *in vivo* degradation rate, which limits their applicability in ALI. Another research team used the azide-acetal linkage as a platform for generating rapidly hydrolyzed (RD)-LNPs to overcome this shortcoming. It was experimentally demonstrated that RD-LNPs designed for the delivery of mRNA to the lungs could rescue mice from ALI by delivering IL-22 mRNA (Zhao et al., 2024).

### 3.4 Mechanism of targeted delivery

The permeability of the alveolar–capillary barrier increases during ALI, leading to an increase in the width of the vascular–endothelial cell gap. Nanomaterials are therefore able to passively accumulate in the alveolar region of this highly permeable area and thus achieve a passive targeting effect via the enhanced permeability and retention (EPR) effect (Figure 4) (Zhang S. et al., 2024). Wang et al. prepared a drug delivery platform based on triangular DNA origami modified with R9 peptide and demonstrated the ability of this nanomaterial to exert an enhanced passive macrophage-targeting effect in a mouse model of ALI (Wang H. et al., 2024). At the onset of ALI, various biomarkers can be more highly expressed on the surface of endothelial cells, including endothelin-1, L-selectin, E-selectin, and P-selectin (Mokra and Kosutova, 2015; Vassiliou et al., 2020). Nanomaterials are capable of targeting these biomarkers that exhibit increased expression and can thus achieve active targeting by ligand-receptor interactions. Li et al. developed a lung-targeted drug delivery system that targeted intercellular adhesion molecule (ICAM)-1 and delivered simvastatin for the treatment of ALI (Figure 4). This system exhibited good lung-targeting properties in mice with lipopolysaccharide (LPS)-induced ALI and was effective in inhibiting the expression of TNF-α and IL-6 and the infiltration of inflammatory cells. This nano-delivery system was shown to significantly improve the histological condition by hematoxylin-eosin staining, which demonstrated that the nanomaterial can treat ALI via this active targeting mechanism mediated by ICAM-1 recognition (Li et al., 2017). A team designed lung-targeted DEX-loaded nanostructured lipid carriers with the same target. These nanocarriers, which were modified with anti-ICAM-1 antibody, were demonstrated to significantly reduce the infiltration of inflammatory cells in the lung and the production of the proinflammatory cytokines TNF-α and IL-6. They also improved the histological condition in a mouse model of LPS-induced ALI (Li et al., 2018). Furthermore, biomimetic strategies have emerged, such as Cell-derived biomimetic nanoparticle. They have characteristics such as low immunogenicity, long circulation time, and strong targeting. Gao et al. conducted a detailed analysis of the current research progress on cell membrane-based biomimetic technology and extracellular vesicle (EV)-based biomimetic nanotechnology in acute lung injury (ALI), and explored their clinical feasibility. Clinical trials involving EVs are currently underway, indicating that biomimetic nanoparticles offer a promising platform for the treatment of ALI (Gao et al., 2024). Another innovative approach utilizes mesenchymal stem cells and their derived exosomes as nanoparticles that can convey most of the biological effects and therapeutic benefits of their source cells, demonstrating high compatibility with damaged lung tissue and targeted accumulation (Zhu et al., 2013). Moreover, responsive targeting systems, including ROS-responsive polymers and pH-sensitive micelles, have allowed for drug release to be selectively triggered in the inflammatory microenvironment. Zhai et al. developed ROS-responsive polysulfone nanoparticles loaded with dexamethasone (PTKNPs@Dex), demonstrating that PTKNPs@Dex can accumulate at sites of pulmonary inflammation and rapidly release the encapsulated payloads, exerting a responsive targeted effect in the treatment of ALI (Zhai et al., 2022).

## 4 Progress of research on nanocarriers for the treatment of ALI

An excessive inflammatory response and lung tissue damage are important features of ALI, and oxidative stress is a principal cause of ALI. An increasing number of studies have shown that nanocarriers have important roles in anti-inflammatory and antioxidant therapies (Lang et al., 2020). Owing to the limited availability of clinical drugs for the treatment of ALI, research on the use of nanocarriers for the treatment of ALI is gradually intensifying. Common types of nanocarriers include LNPs, polymer NPs, and inorganic nanomaterials. Next, new findings with regard to the use of nanocarriers in the treatment of ALI are introduced.

### 4.1 Liposomes

**FIGURE 5:**
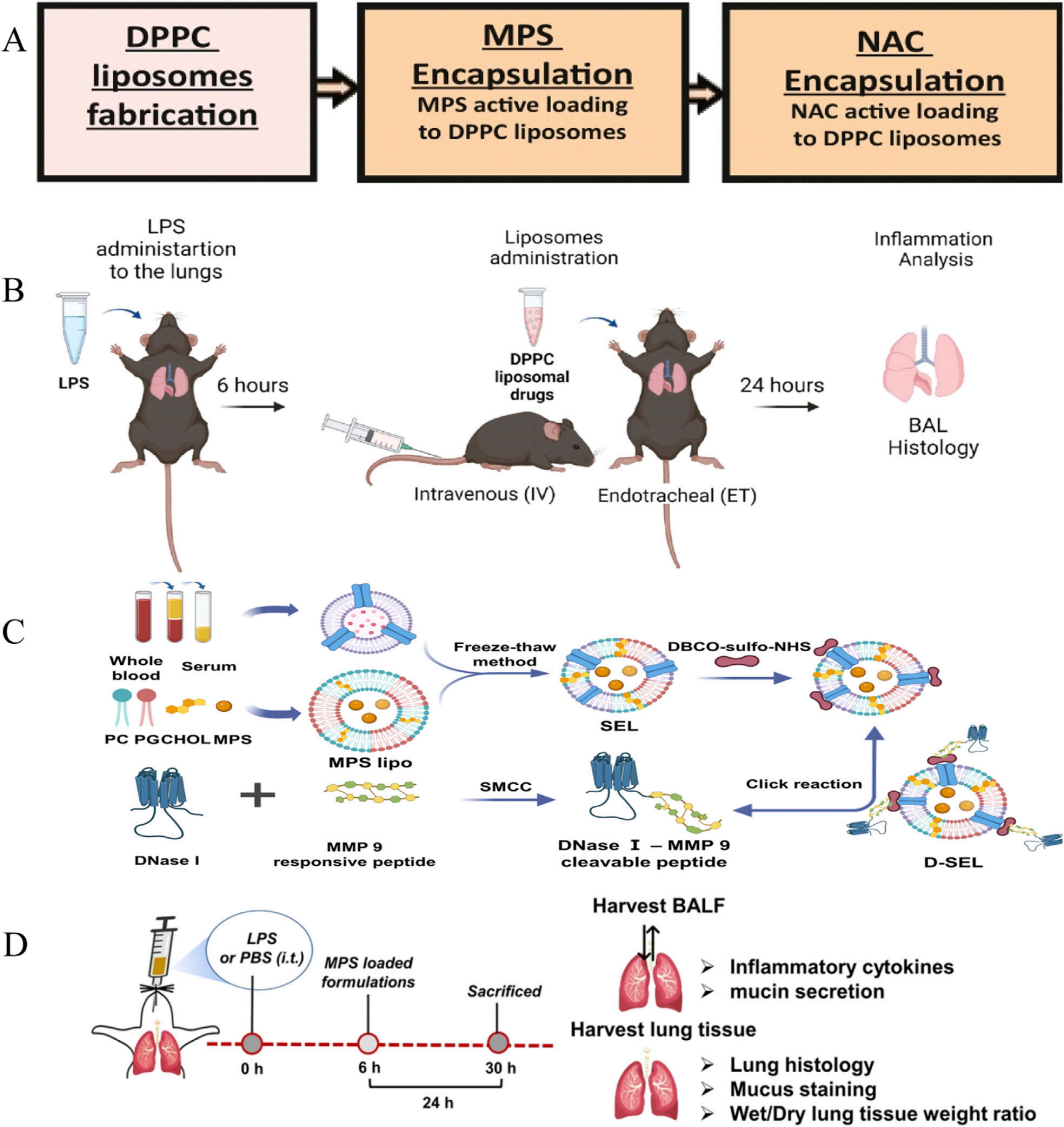
Liposomes therapy for ALI. **(A)** MPS and NAC preparation. **(B)** Flow chart of DPPC lipid drug experiment (Arber Raviv et al., 2022) Copyright (2022), with permission from Elsevier. **(C)** D-SEL design, preparation process (Liu et al., 2023) Copyright (2023) American Chemical Society. **(D)** D-SEL *in vivo* procedure.

Lipid nanocarriers are mainly amphiphilic or hydrophobic molecules composed of phospholipids, cholesterol, fat-soluble drugs, and other auxiliary lipids (Figure 1) (Gordillo-Galeano and Mora-Huertas, 2018). Owing to their simple processing, high biocompatibility, high bioavailability, etc. (Sercombe et al., 2015; Fonseca-Santos et al., 2015; Mitchell et al., 2020), they make efficient drug delivery systems available. The main types of Lipid nanoparticle (LNPs) include liposomes, solid lipid carriers, nanostructured lipid carriers, and nanoemulsions. Bian et al. summarized the characteristics of various lipid nanomaterials used for the treatment of ALI. These included 1,2-Dipalmitoyl-sn-glycero-3-phosphocholine (DPPC) used for the preparation of drug formulations containing DEX and α-tocopherol/glutathione and other lipid components used for the preparation of different medications, e.g., cholesterol, soybean lecithin, and distearoylphosphatidylethanolamine-polyethylene glycol (PEG) (Figure 5A) (Suntres and Shek, 2000; Suntres and Shek, 1996; Hettiarachchi et al., 2019; Chen et al., 2013). The efficacy of LNPs in the treatment of ALI was demonstrated by determining their effects on lung weight, the lung index, and levels of proinflammatory cytokines (e.g., TNF-α, IL-1β, and IL-6), neutrophil elastase, and myeloperoxidase (MPO) and assessing their therapeutic effects in different mouse models (Bian et al., 2021b). Ravivi et al. similarly developed liposomes with DPPC as the main lipid and with sizes of 100 nm for pulmonary drug delivery for the treatment of ARDS. The loading efficiency of drugs in these liposomes reached 98% for methylprednisolone (a steroid) and 92% for NAC (a mucolytic agent). In cell experiments, a reduction in secretion of TNF-α and NO was observed in LPS-stimulated RAW 264.7 macrophages treated with the liposomes. In C57BL/6 mice used as a model of LPS-induced lung inflammation, the therapeutic efficacy of the liposomes in reducing inflammation and secretion of the cytokines TNF-α, IL-6, and IL-1β was observed to be better than that of the free drug administered by the intravenous and endotracheal routes (Figure 5B) (Arber Raviv et al., 2022). Inhalable nanomedicine delivery systems encapsulate drugs within nanoscale carriers and deliver them via inhalation to specific regions or areas of the lungs, particularly those with affected or diseased cells (Kuzmov and Minko, 2015). Compared to traditional treatment methods, inhalable nanomedicine delivery systems can penetrate the mucus barrier, deposit precisely at lung lesion sites, prolong drug retention time, and reduce administration efficiency. Currently, this delivery system is widely used in the treatment of lung cancer, chronic obstructive pulmonary disease, asthma, and other diseases (Jin Z. et al., 2023; Gupta et al., 2022; Anderson et al., 2020; Goswami et al., 2025). Liu et al. developed an inhalable nanoplatform for sequential drug release referred to as D-SEL. This was formed by attachment of a peptide cleavable by matrix metalloproteinase-9 to serum exosomes and liposomes and subsequent encapsulation of methylprednisolone sodium succinate (MPS) (Figure 5C). It was found that treatment with MPS/D-SEL significantly inhibited activation of neutrophils and macrophages, reduced the number of neutrophils, and promoted the polarization of M2-type macrophages in a mouse model of LPS-induced ALI. It also effectively inhibited the expression of proinflammatory cytokines (TNF-α, IL-1β, and IL-6), increased the level of production of anti-inflammatory cytokines (IL-4 and IL-10), and alleviated pathological disorders in the lungs (Figure 5D) (Liu et al., 2023).

### 4.2 Polymers

**FIGURE 6:**
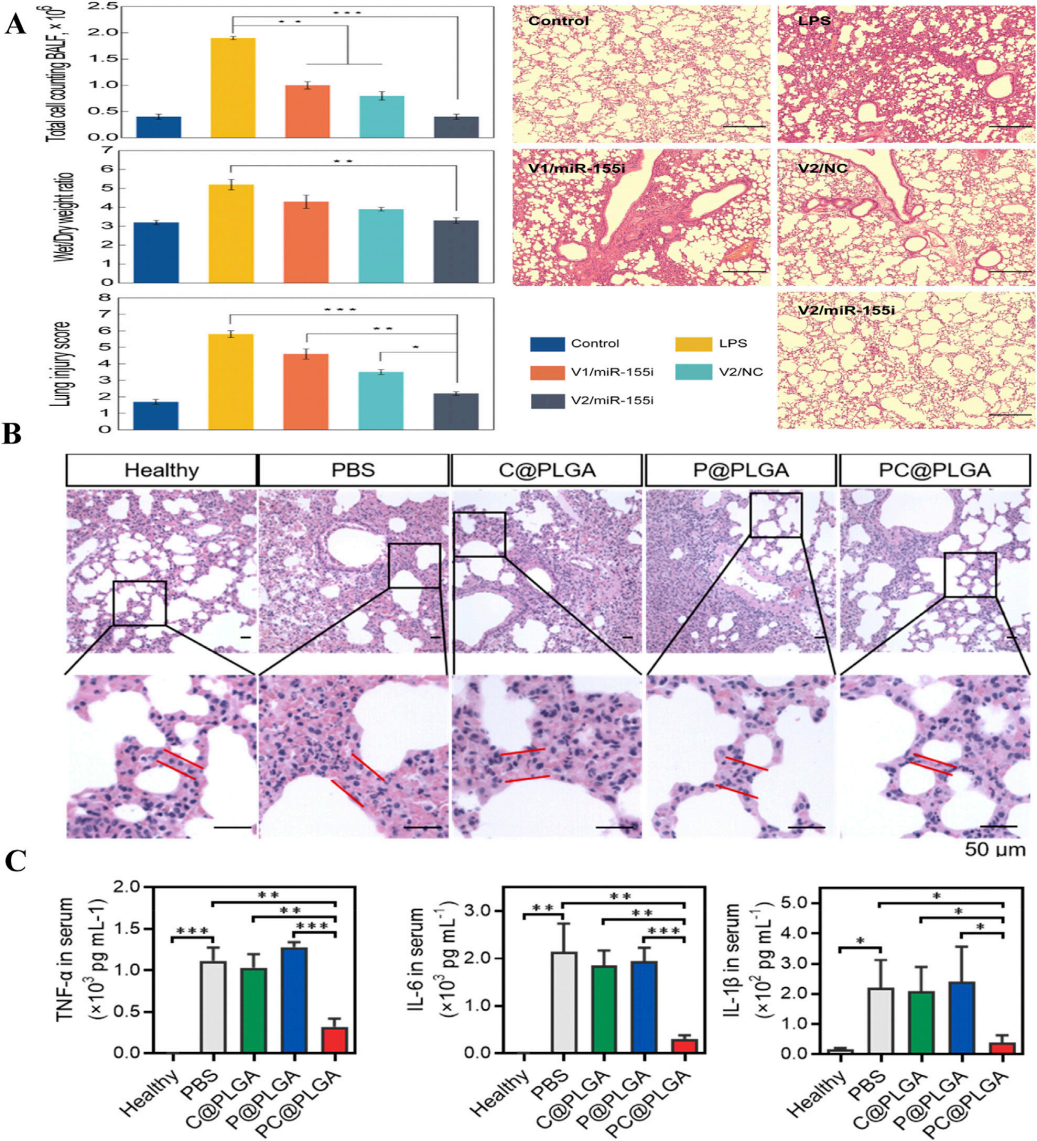
Polymer therapy for ALI. **(A)** Comparison of the effects of different treatments on lung injury (Li et al., 2021) Copyright (2021) American Chemical Society. **(B)** Histopathological observations of PC@PLGA in an inflammatory model of lung injury. **(C)** Analysis of PC@PLGA levels of inflammatory factors (TNF-α, IL-6, IL-1β) in an inflammatory model of lung injury (Chen et al., 2023) Copyright (2023) American Chemical Society.

Polymeric nanocarrier systems are constructed from natural or synthetic polymers formed from either monomeric units or pre-existing polymers (Figure 1). Various synthesis techniques are available for creating polymeric nanomaterials, including emulsification (via solvent displacement or diffusion), nanoprecipitation, ionic gelation, and microfluidic methods (Kurd et al., 2019; Sawasdee et al., 2018). Polymeric nanomaterials can efficiently carry hydrophobic and hydrophilic drug molecules as a result of their flexible drug delivery capabilities. Consequently, they are extensively employed in domains such as pharmaceutical delivery and genetic delivery. Among these nanomaterials, poly (lactic-co-glycolic) acid (PLGA)-based polymer NPs have been proven to exhibit good drug release tunability, which is a popular area for polymer NPs in terms of research and applications (Jha and Mayanovic, 2023). Recent studies have found that PLGA-mediated stimulation of aging signaling triggers precise macrophage-mediated clearance of inflammatory neutrophils, which can ultimately have the effect of reducing inflammation. Chen et al. developed PC@PLGA polymeric nanoparticles by encapsulating PLGA with platelet-derived vesicle membranes (P) and calmodulin-expressing membranes (C), enabling specific targeting of activated neutrophils and deceiving macrophages into recognizing them as “aging” neutrophils. In an LPS-induced ALI mouse model, these nanoparticles effectively targeted activated neutrophils promoted macrophage-mediated programmed cell removal, and reduced inflammation and tissue damage (Chen et al., 2023). In addition, dendritic macromolecules, with their large internal cavities and dense surface active functional groups, are able to improve bioavailability and biocompatibility and have been studied extensively in ALI (Nikzamir et al., 2021; Albr et al., 2008). Polyamide-amine (PAMAM) dendrimer is one of the most intensively studied dendrimers. Fifth-generation (G5) PAMAM dendrimer was used as the basic platform, which was modified with DEX and PEG on its surface, and gold nanoparticles (GNPs) were encapsulated in the internal cavity to form a nanocarrier with the composition (Au^0^)_25_-G5NH_2_-(PEG-DEX) (denoted by V2). The nanocarrier was then electrostatically bound to a microRNA-155 inhibitor of an anti-inflammatory gene (miR-155i) to form a nanocomplex (V2/miR-155i) (Figure 6A). *In vitro* studies with mouse alveolar macrophages showed that the nanocomplex exhibited low cytotoxicity and efficiently co-delivered miR-155i and DEX, suppressing TNF-α, IL-1β, and IL-6 mRNA expression (Figure 6C). In an LPS-induced ALI mouse model, airway nebulization of the nanocomplex significantly inhibited proinflammatory factors and improved lung repair, outperforming single-modality chemotherapy and gene therapy, as confirmed by histopathological analysis (Figure 6B) (Li et al., 2021).

### 4.3 Inorganics

**FIGURE 7:**
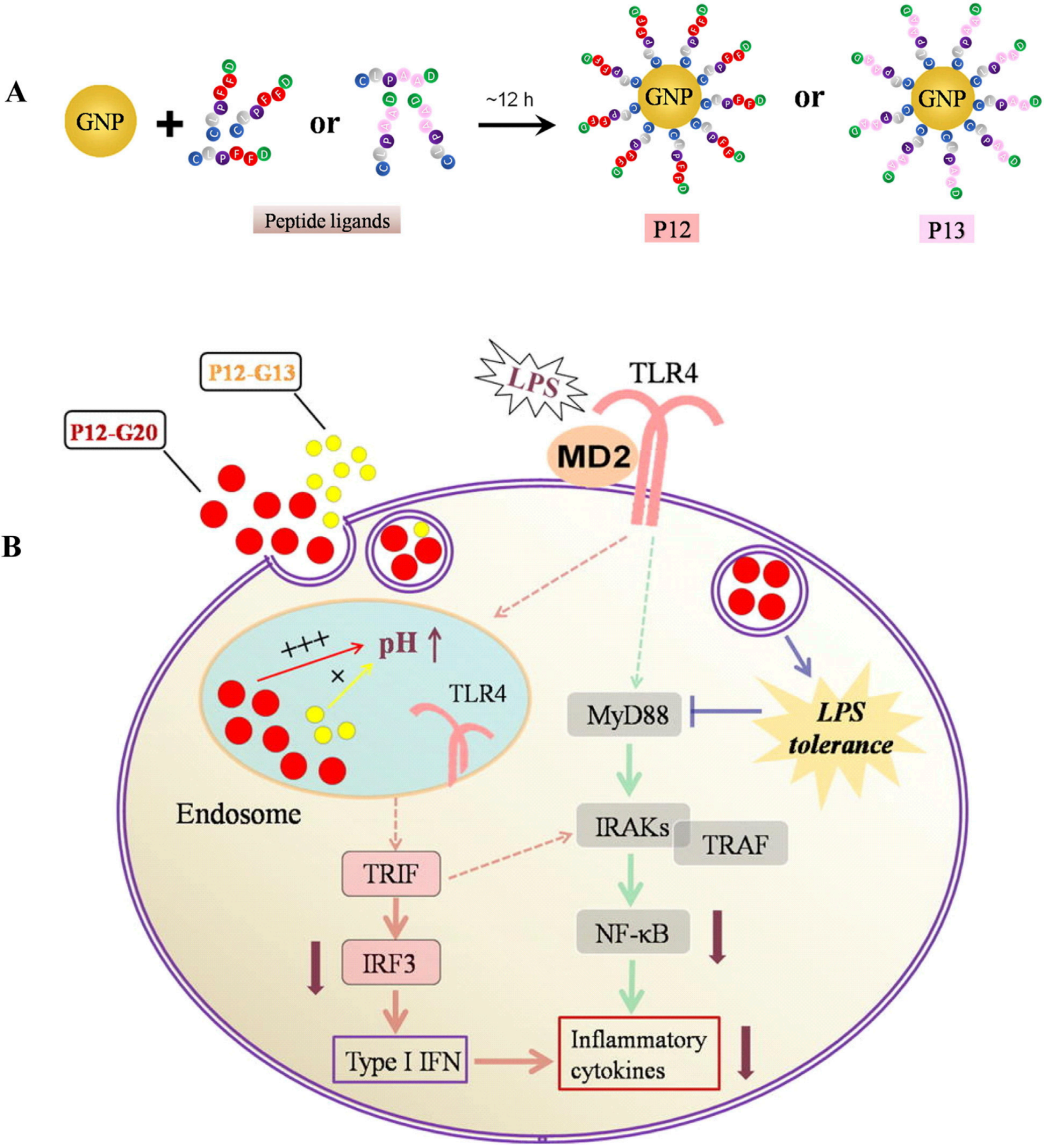
Inorganic nanomedicine. **(A)** Fabrication and characterization of bare GNPs and peptide-GNP hybrids with different sizes. **(B)** Mechanism of enhanced inhibitory activity of hybrid P12 (G20) on TLR4 activation (Gao et al., 2019) Copyright (2019), with permission from Elsevier.

Inorganic nanomaterials have been widely studied due to their stable structures, high drug loading rates, and easy surface modification (Figure 1) (Cho et al., 2010). Studies have shown that these materials hold significant potential in the treatment of ALI (Bian et al., 2021b). GNPs, which are a popular subject of research, have been shown to alleviate ALI by inhibiting inflammatory signaling pathways and scavenging ROS (dos Santos Haupenthal et al., 2020). The cumulative toxicity exhibited *in vivo*, stemming from their prolonged retention within the liver and spleen, coupled with their inherently poor biocompatibility, presents challenges that necessitate further research. Consequently, inorganic peptides have been employed for the modification of GNPs, which has enhanced their biocompatibility and alleviated their associated toxic side effects (Singh et al., 2018). Gao et al. designed a unique class of peptide-GNP hybrids that act as potent nano-inhibitors of TLR4 signaling by modulating the endosomal acidification process. It was found that the size of the NPs was a significant factor in their inhibitory effect on TLR4 (Figure 7B). In particular, a peptide-GNP hybrid with a 20 nm GNP core (P12 (G20)) exhibited the strongest inhibitory activity in THP-1 cell-derived macrophages (Figure 7A). In a mouse model of LPS-induced ALI, P12 (G20) was more effective than P12 (G13) (with a 13 nm GNP core) in prolonging survival time, reducing lung inflammation, and alleviating alveolar injury (Gao et al., 2019). Molybdenum NPs are also promising for scavenging ROS, according to new research findings. Yan et al. developed a functional nanomaterial, namely, molybdenum nanodots (MNDs), with a size of approximately 5 nm by ultrasonic stripping. Their ability to scavenge ROS was assessed by assaying cellular activity in RAW 264.7 cells (a mouse macrophage cell line) and MLE-12 cells (a mouse lung epithelial cell line). It was found that MNDs were able to induce a significant increase in the activity of RAW 264.7 and MLE-12 cells. Intracellular ROS levels were found to be significantly reduced after fluorescence staining. This demonstrated that MNDs were able to limit oxidative-stress-induced damage to RAW 264.7 and MLE-12 cells by scavenging ROS. In a mouse model of LPS-induced ALI, after injection by tracheal drip of different doses of MNDs, bronchoalveolar lavage fluid (BALF) and lung tissues were taken. The levels of ROS in the lung tissues of the mice were determined by dihydroethidium staining, which revealed that the levels of ROS in the lung tissues of mice treated with MNDs were significantly reduced. In addition, it was found that MNDs reduced MPO levels in BALF and decreased the numbers of monocytes/macrophages and neutrophils in lung tissue. Lung histopathological examination showed that MNDs were able to reduce lung tissue injury, which proved that MNDs can exert anti-inflammatory and antioxidant effects and thus reduce ALI (Yan et al., 2023).

## 5 Recent developments in NP-NMs for treating ALI

Natural products, predominantly sourced from herbs, inherently possess biocompatibility, thereby significantly reducing adverse effects when incorporated into nanomedicines. Their biodegradable nature ensures they decompose within the body, thus diminishing their potential for toxicity (Wang et al.). Owing to their complex and diverse chemical structures, natural products can interact with nanomedicines to target multiple pathways and injury sites with precision, which improves the accuracy and effectiveness of treatment (Wang et al.; Atanasov et al., 2021). In addition, natural products can be integrated with nanocarriers to enable controlled release in response to specific stimuli, such as temperature, enzymes, or pH (Xu et al., 2015). Natural products also possess inherent therapeutic properties, including anti-inflammatory, antimicrobial, antioxidant, and anticancer activities (Fernandes et al., 2023; Guo et al., 2024; Akbari et al., 2022; Hashem et al., 2022). However, their unique structures and poor solubility limit their therapeutic potential and bioavailability. By combining natural products with nanocarriers, their solubility and bioavailability can be enhanced, which can maximize their efficacy. This approach offers a promising strategy for the treatment of ALI. The following sections discuss the use of various nanocarrier-based natural products for treating ALI.

### 5.1 Polyphenols

Polyphenols are a class of plant-derived antioxidants that are structurally based on the benzene ring and are found in everyday human foods in the form of glycosides or glycosidic elements.

#### 5.1.1 Curcumin

Curcumin, a hydrophobic polyphenol derived from the roots of turmeric (Curcuma longa), exerts anti-inflammatory, antioxidant, and antiapoptotic effects by modulating molecular targets through multiple signaling pathways (Huang et al., 2018; Gupta et al., 2013; Shakeri and Boskabady, 2017; Hu et al., 2017; Xu and Zhu, 2017). Leng et al. developed an amphiphilic multifunctional poly (citric acid-polyethylene glycol-curcumin) (PCGC) nano-oligomer using citric acid, curcumin, and PEG via a one-pot thermal polymerization synthesis strategy. PCGC was found to inhibit the expression of TNF-α, IL-6, NF-κB, and IL-1β. The incorporation of curcumin enhanced the ability of PCGC to inhibit the expression of proinflammatory factors. PCGC can also effectively inhibit pulmonary edema and reduce infiltration of bronchial cells and can thereby alleviate ALI (Leng et al., 2024). Additional studies have shown that curcumin-loaded tyramine-bearing sodium dimethylphosphonate-modified amphiphilic phosphorus dendron (C11G3-TBP@Cur) nanomicelles have good biosafety and stable loading capacity. In addition, C11G3-TBP@Cur inhibited the expression of TNF-α, IL-1β, IL-6, and MPO while promoting the secretion of anti-inflammatory cytokines (arginase-1, IL-10, and CD206). This indicated that C11G3-TBP@Cur could promote the repolarization of alveolar macrophages from the M1 type to the anti-inflammatory M2 type. Histopathological findings indicated that C11G3-TBP@Cur was able to promote the repair of lung tissue injury (Li et al., 2022). In addition, inorganic NPs have been used in combination with curcumin. Yuan et al. synthesized iron-curcumin nanoparticles (Fe-Cur NPs) and demonstrated that Fe-Cur NPs significantly reduced the levels of the proinflammatory factors TNF-α, IL-1β, and IL-6 in both *in vivo* and *ex vivo* experiments. In addition, Fe-Cur was shown to inhibit NLR family pyrin-domain-containing 3 (NLRP3) and NF-κB and thereby exert anti-inflammatory effects. This suggests that Fe-Cur NPs may exert anti-inflammatory effects via multiple pathways. In a mouse model, Fe-Cur NPs exhibited higher distribution levels in lung tissues than in the heart, liver, and kidneys, which suggested precise targeting of affected areas. Moreover, Fe-Cur NPs possess ROS-scavenging abilities, which contribute to the treatment of ALI. Furthermore, Fe-Cur NPs also reduced the numbers of macrophages and CD3^+^CD45^+^ T cells, which served to inhibit inflammatory cytokine storms (Yuan et al., 2021). Piao et al. prepared curcumin-loaded glycyrrhetinic acid nanoparticles (GA-Cur) to address the low bioavailability of curcumin due to its hydrophobicity. They observed that GA-Cur inhibited hemolysis of monocytes and lung infiltration more efficiently and reduced the levels of proinflammatory cytokines in comparison with other treatments (Piao et al., 2022). Sun et al. prepared curcumin-loaded ROS-responsive bovine serum albumin-coated hollow mesoporous silica nanoparticles (Cur@HMSN-BSA), which provide a new therapeutic option for PM2.5-induced ALI (Sun et al., 2023). Inhalable arginine-modified chitosan nanocrystals containing curcumin (Arg-CS-Cur) were prepared by Wu et al. Arg-CS-Cur was found to exhibit higher uptake by M1 macrophages *in vitro* and to inhibit TNF-α and IL-6 effectively. In a rat model of ALI, lung tissue damage was reduced after treatment with the nanocrystals (Wu et al., 2024a). Su et al. developed a highly crosslinked curcumin-containing polyphosphazene nanodrug (PHCH) for the targeted delivery and inflammation-responsive release of curcumin for the treatment of ALI. It was experimentally demonstrated that this nanomedicine was able to downregulate the expression of key proinflammatory cytokines (e.g., TNF-α, IL-1β, and IL-8) and inhibit the activation of the NLRP3 inflammasome and NF-κB signaling pathway and thus exert anti-inflammatory activity. In a mouse model of ALI, PHCH exhibited effective ROS-scavenging ability, which is beneficial for the treatment of ALI (Su et al., 2023).

#### 5.1.2 Resveratrol

Resveratrol (RSV), naturally occurring in various foods like blueberries, mulberries, and grapes, has been proven to possess significant biological functions in the treatment of inflammatory diseases. Oliveira et al. prepared resveratrol-containing polymer nanocapsules (RSV-LNCs) using interfacial polymer deposition and demonstrated their ability to inhibit leukocytes and reduce the expression of transcription factors related to the inflammatory response. By evaluating the anti-inflammatory and antioxidant effects of RSV-LNCs 6 h after exposure to LPS in a mouse model of ALI, they confirmed the potential of RSV-LNCs to reduce lung inflammation and improve lung function to treat ALI. RSV levels in mouse tissues were also found to be higher than after the administration of the free form of RSV, which demonstrated the enhanced bioavailability of RSV in the RSV-LNC form (de Oliveira et al., 2019). In another study, PLGA NPs were combined with platelet membrane vesicles (PMs) to prepare a new type of biomimetic NPs referred to as PM@Cur-RV NPs, which could deliver curcumin and RSV in a highly targeted manner. In a mouse model of ALI, inhalation of PM@Cur-RV NPs reduced pulmonary vascular permeability and the proinflammatory cytokine load and thereby effectively inhibited pulmonary vascular injury. In addition, it reduced the level of histone acetylation in macrophages and promoted polarization of macrophages toward the M2 phenotype, thereby alleviating ALI (Jin et al., 2022). Wang et al. prepared an inhalable RSV formulation (referred to as RES-β-CD) consisting of an inclusion complex formed by RSV and β-cyclodextrin for preventing ALI induced by zinc chloride smoke. RES-β-CD exhibited low cytotoxicity and, in a mouse model of ALI, it was able to inhibit the expression of TNF-α, IL-1β, STAT3, and GATA3 and upregulate T-box transcription factor and forkhead box P3 to achieve alleviation of inflammation and apoptosis. It thus provides a new idea for the treatment of smoke-induced ALI (Wang et al., 2022).

#### 5.1.3 Paeonol

Paeonol (PAE), a naturally occurring active compound derived from the root bark of the peony plant, exhibits diverse pharmacological activities and is a quintessential member of the anti-inflammatory class of active ingredients (Wang et al., 2020). Li et al. used incubation to load PAE into a γ-cyclodextrin metal-organic framework (CD-MOF) with a respirable particle size and mix it with lactose to prepare a dry powder inhaler (DPI). The physicochemical properties of PAE-CD-MOF were observed using scanning electron microscopy, powder X-ray diffraction analysis, thermal analysis, and hot-stage microscopy. The *in vitro* release behavior of PAE from CD-MOF was observed, and it was found that the release of PAE was significantly accelerated in simulated lung fluid. Moreover, via *in vitro* cell experiments, CD-MOF was found to increase the cellular permeability of PAE in A549 lung adenocarcinoma cells, which suggested that CD-MOF may promote the uptake of PAE in the lung. A healthy rat model was established, and it was demonstrated that administration by inhalation significantly increased the bioavailability of PAE in comparison with oral administration. In a rat model of ALI, histopathological examination and measurement of serum levels of proinflammatory factors confirmed that the PAE-CD-MOF DPI reduced lung inflammation and thereby alleviated lung injury (Li et al., 2020).

#### 5.1.4 Catechin

Catechin belongs to a class of flavanols found in tea and is an important secondary metabolite. Jin et al. prepared a novel nanomicellar complex (Ac/Pc) by binding catechin to a cationic lipid polymer (polyethyleneimine-cholesterol, Pc) and serum albumin (Ac). The nanomicelles were evaluated for their capacity to selectively induce apoptosis in A549 lung cancer cells at low concentrations. The nanomicelles induced a rise in ROS levels and stimulated the activity of caspase-3 in lung cancer cells, thus promoting apoptosis. In in vivo real-time fluorescence imaging using cyanine 5-labeled nanomicelles injected into mice, the cationic polymeric liposomes (Pc) were demonstrated to have lung-targeting efficacy, while albumin (Ac) contributed to prolonged retention of the complex *in vivo*. These results suggest that the nanomicellar complex possesses lung-targeting capability and can effectively deliver drugs, which provides a promising strategy for treating ALI (Jin M. et al., 2023).

### 5.2 Flavonoids

Flavonoids are a class of natural substances widely found in nature, with 2-phenylchromone as the parent structure, usually in the form of free glycosides or combined with sugar to form glycosides, with a wide range of biological activities and pharmacological effects.

#### 5.2.1 Quercetin

Quercetin is a flavonol widely found in the bark, flowers, leaves, seeds, and fruits of a wide variety of plants. Quercetin has been demonstrated to have various biological activities, including anti-inflammatory, antioxidant, and immunomodulatory activities (Chen J. et al., 2022; Yang et al., 2020). Chen et al. prepared an inhalable quercetin-alginate nanogel (QU-Nanogel) using emulsion polymerization. The “substance-drug” complex formed between the two components was stabilized via intermolecular hydrogen bonds, forming a synergistically developed water-soluble nanogel system. Experiments on A549 cells examined the toxicity of QU-Nanogel and revealed its inhibitory effect on ROS. In a rat model of ALI, histological analysis after administration by ultrasonic nebulization showed strong fluorescence intensity in the rat lung tissues, which indicated good lung targeting and renal excretion and confirmed the safety of QU-Nanogel. Levels of three proinflammatory cytokines (TNF-α, IL-6, and IL-1β) in lung tissues were measured, and the expression of these cytokines was significantly reduced following treatment with QU-Nanogel. These findings suggest that QU-Nanogel can downregulate the mRNA and protein expression of proinflammatory factors via ultrasonic nebulization and inhalation and thereby alleviate lung inflammation (Chen Y.-B. et al., 2022). Zhang et al. prepared coordination polymer nanoparticles (MCQ/R NPs) using D-mannitol, cerium ions, quercetin, and rutin. In a mouse model of LPS-induced ALI, treatment with MCQ/R NPs significantly reduced the lung wet weight/dry weight ratio and the total cell count and number of inflammatory cells (neutrophils and macrophages) in BALF. Serum levels of IL-6, IL-1β, and TNF-α and the mRNA expression of TLR4 and NLRP3 were also reduced. Histopathological examination demonstrated that MCQ/R NPs reduced the extent of inflammatory infiltration and interstitial edema in mice, which provides an idea for the treatment of ALI (Zhang Y. et al., 2024).

#### 5.2.2 Pachypodol

Pachypodol is a significant secondary metabolite that is widely present in nature and is classified as a plant flavonoid. Sun et al. encapsulated the hydrophobic flavonol pachypodol in a liposome (Pac-lipo) and demonstrated that *in vitro* Pac-lipo exhibited anti-inflammatory and protective effects on the endothelial and epithelial barriers of macrophages and endothelial cells exposed to LPS. In addition, in a mouse model of LPS-induced ALI, Pac-lipo inhibited the expression of cytokines including TNF-α, IL-6, IL-1β, and inducible nitric oxide synthase (iNOS) in lung tissues. It also repaired the lung epithelial barrier and vascular endothelial barrier and inhibited the activation of the TLR4-MyD88-NF-κB/MAPK signaling pathway, and it thereby reduced lung injury (Sun et al., 2024).

#### 5.2.3 Hesperidin

Hesperidin, a natural flavonoid found in citrus fruits and vegetables, has demonstrated the ability to treat Acute Lung Injury (ALI) in an experimental mouse model (Dong et al., 2020; de Souza et al., 2024). Jin et al. prepared chitosan NPs loaded with hesperidin (HPD/NPs) for nasal delivery to inflamed lungs. In a mouse model of LPS-induced ALI, IL-1β and IL-6 levels in peripheral blood, together with TNF-α, IL-17, and NO levels in BALF, were measured to assess the inflammatory response. The results confirmed that HPD/NPs reduced the levels of the proinflammatory cytokines and alleviated lung injury more effectively than free hesperidin. Pulmonary vascular permeability was assessed using the Evans blue-albumin extravasation assay, and expression levels of IL-1β and caspase-1 were determined by immunohistochemical staining of lung tissues. The findings showed that HPD/NPs were effective in reducing pulmonary vascular permeability and inhibiting cellular sepsis, which suggested that they hold potential for the treatment of ALI (Jin et al., 2021).

#### 5.2.4 Luteolin

Luteolin is a flavonoid commonly found in fruits, vegetables, flowers, and herbs. Luteolin has exhibited superior therapeutic efficacy in experimental models of ALI (Xie et al., 2021; Zhang et al., 2021). Gu et al. synthesized cerium ion-luteolin protein nanocomplexes (CeLutNCs) by coordinating cerium ions with luteolin. *In vitro* experiments demonstrated that CeLutNCs effectively scavenged various ROS, including H_2_O_2_, O_2_^−^, ·OH, DPPH·, and ABTS^+^, and exhibited favorable cytoprotective effects in RAW 264.7 mouse macrophages. In a mouse model of ALI, CeLutNCs also exhibited therapeutic effects, reducing inflammatory responses and histopathological alterations (Gu et al., 2024).

#### 5.2.5 Baicalin

Baicalin is one of the main active ingredients extracted from the Chinese medicine baical skullcap root, which has anti-inflammatory properties (Hu et al., 2021; Fu et al., 2021). Yu et al. prepared a new drug delivery system, namely, baicalin liposome (BA-LP), which overcame the defect of the low solubility of baicalin. In a mouse model of LPS-induced ALI, BA-LP was found to reduce the lung wet weight/dry weight ratio, reduce the lung injury score, and inhibit the expression of proinflammatory factors (TNF-α and IL-1β) in BALF. It also exerted an anti-inflammatory effect by inhibiting the TLR4-NF-κBp65 and c-Jun N-terminal kinase-extracellular signal-regulated kinase signaling pathways. BA-LP thus provides a new option for the treatment of ALI (Long et al., 2020).

### 5.3 Alkaloids

Alkaloids are a class of nitrogen-containing alkaline organic compounds that are widely found in plants and have a variety of biological activities such as antimicrobial, anti-inflammatory, antioxidant, antiviral, immunomodulatory and analgesic.

#### 5.3.1 Ligustrazine

Ligustrazine is an alkaloid extracted from the traditional Chinese medicinal herb chuanxiong. He et al. synthesized a novel reactive oxygen-sensitive carrier in the form of a covalent cyclodextrin framework (OC-COF), which was based on a CD-MOF, using oxalyl chloride as a crosslinking agent. A reactive oxygen-sensitive DPI referred to as LIG@OC-COF was developed by loading ligustrazine onto OC-COF. LIG@OC-COF exhibited anti-inflammatory and antioxidant effects at the cellular level *in vitro* and in an animal model of ALI. In addition, combining ligustrazine with nanomaterials enhanced its bioavailability (H et al., 2022).

#### 5.3.2 Capsaicin

Capsaicin is an active compound derived from chili peppers. It has been shown to have great potential in pain, high blood pressure, inflammation, and other conditions (Ferreira et al., 2020; Richards et al., 2012; Singh and Bernstein, 2014). Dynamic development of capsaicin’s anti-inflammatory ability is an effective way to treat ALI. Wang et al. prepared NPs referred to as Fe-CAP NPs containing capsaicin and iron. The anti-inflammatory ability of Fe-CAP NPs was confirmed by cell experiments, which revealed that the expression of TNF-α and iNOS was reduced in LPS-induced RAW 264.7 cells treated with Fe-CAP NPs and that these NPs could regulate the NF-κB signaling pathway. In a mouse model of ALI, Fe-CAP NPs inhibited the expression of IL-6 and iNOS. Via histopathological studies, it was observed that Fe-CAP NPs reduced the leakage of Evans blue in the lung and alleviated lung histopathology in rats and thus exhibited good anti-inflammatory ability, which holds significance for the treatment of ALI (Wang R. et al., 2024).

### 5.4 Terpenoids

Terpenoids are a class of organic compounds formed by the polymerization of isoprene units (C5H8) that are rich in chemical and biological activities such as anti-inflammatory, anti-cancer and neuroprotective effects (Zhao et al., 2022).

#### 5.4.1 Tanshinones

Tanshinones are a class of fat-soluble bioactive compounds extracted from the traditional Chinese medicinal herb Danshen. El-Moslemany et al. prepared a nanoemulsion using a biosurfactant (rhamnolipid) and tea tree oil loaded with tanshinone IIA (TSIIA) via ultrasonication. In a model of LPS-induced ALI, animals treated with the TSIIA nanoemulsion (TSIIA-NE) exhibited significant increases in tidal volume and respiratory rate, a reduction in the lung wet weight/dry weight ratio, and an improvement in arterial blood gas levels. In addition, histopathological examination of the lung and biochemical analysis of various biomarkers suggested that TSIIA-NE exhibited antioxidant and anti-inflammatory effects and thereby alleviated symptoms of LPS-induced ALI (El-Moslemany et al., 2022).

### 5.5 Glycosides

Glycosides are natural products synthesized from a variety of natural plants, with anti-inflammatory, anti-tumor, antioxidant and other effects.

#### 5.5.1 Ginsenoside

Ginsenoside Rb1 is a principal bioactive component of ginseng (Zhang et al., 2023). Wu et al. prepared novel bionanoparticles (R@ZQC NPs) loaded with ginsenoside Rb1, utilizing a metal-organic framework (zeolitic imidazolate framework-8 NPs) as a carrier. These particles encapsulated ginsenoside Rb1 internally and were coated externally with a quaternary chitosan layer and a macrophage membrane. Experimental results indicated that R@ZQC NPs enhanced mitochondrial function and reduced oxidative stress by activating or directly binding to AMP-activated protein kinase, inhibited apoptosis in alveolar macrophages, and thus mitigated ALI. In a mouse model of ALI, tail vein administration of R@ZQC NPs increased the survival rate of mice with sepsis-induced ALI and alleviated histopathological damage to the structure of the lungs. These results suggest a promising therapeutic approach for addressing ALI caused by sepsis (Wu et al., 2024b).

### 5.6 Triterpenoids

Triterpenoids are terpenoids with a basic nucleus consisting of 30 carbon atoms that have a wide range of biological activities including anti-inflammatory, antioxidant, antitumor and antifibrotic.

#### 5.6.1 Celastrol

Celastrol is derived from the root bark of the traditional Chinese medicine Common Threewingnut Root and is a natural product with a variety of biological activities. Yao et al. developed a mannose-modified drug delivery system for targeted delivery of celastrol to alveolar macrophages. Celastrol nanoparticles (Cel-NPs) were synthesized using emulsification and evaporation techniques. *In vitro* studies demonstrated that mannose-modified Cel-NPs significantly enhanced the delivery of celastrol to inflammatory macrophages and exhibited strong biocompatibility. In a mouse model of ALI, Cel-NPs effectively inhibited infiltration of inflammatory cells, reduced lung edema, and suppressed LPS-induced lung inflammation and cytokine storms, which suggested that they hold promise for treating ALI (Yao et al., 2024).

### 5.7 Coumarins

Coumarins are a group of secondary metabolites widely found in plants with different biological activities such as anti-inflammatory, antiviral and bacteriostatic effects using phenanthrene α-pyrone as the parent nucleus.

#### 5.7.1 Bergapten

Bergamot lactone (bergapten) is a coumarin analog widely found in medicinal plants such as Buddha’s hand and Dahurian angelica root (B et al., 2016; Li et al., 2011; Murray and Wynn, 2011). It has various pharmacological activities such as anti-inflammatory, antitumor, and antioxidative effects (Luo et al., 2023; Adakudugu et al., 2020). Liao et al. integrated bergapten and DPPC liposomes to develop a biologically active lung-targeted lipid nanomedicine named Ber-lipo. Ber-lipo was found to exhibit good biocompatibility, low cytotoxicity, and lung-targeting properties and repaired the epithelial and endothelial barriers. Immunofluorescence analysis showed that Ber-lipo decreased the proportion of M1-type macrophages and increased the proportion of M2-type macrophages in LPS-stimulated RAW 264.7 cells, thereby maintaining the M1/M2 balance. In a mouse model of LPS-induced ALI, Ber-lipo significantly reduced body weight loss and the lung index and alleviated pulmonary edema. In addition, it was found that Ber-lipo could effectively inhibit the activation of the TLR4/MyD88/NF-κB proinflammatory pathway and thereby alleviate ALI (Liao et al., 2025) (Table 2).

**TABLE 2:** Summary of recent advances in the treatment of ALI with NP-NMs.

Category	Phytochemical	Natural-product-based nanomaterial	Therapeutic effect	Ref.
Polyphenols	Curcumin (Cur)	PCGC Fe-Cur	TNF-α, IL-1β, IL-6, NF-κB↓; lung volume↓ TNF-α, IL-1β, IL-6↓; Ca^2+^↓; NLRP3, NF-κB↓; PIP2↓; lung index↓; spirometry↑	Leng et al. (2024) (Yuan et al., 2021)
		GA-Cur	TNF-α, IL-1β, IL-6↓; HO-1↑; NF-κB↓	Piao et al. (2022)
	Resveratrol (RSV)	Cur@HMSN-BSA Arg-CS-Cur C11G3-TBP@Cur PHCH RSV-LNCs PM@Cur-RV RES-β-CD	TNF-α, IL-1β, IL-6↓; M1 ↓, M2↑; MDA, MPO↓ TNF-α, IL-6↓; NO↓ TNF-α, IL-1β, IL-6↓; NO, iNOS↓; NF-κB↓; HO-1, SOD-2, NOX-2↓; lung dry weight/wet weight ratio of mice↓; MPO↓ TNF-α, IL-1β, IL-8↓; NLRP3↓; NF-κB↓ ERK/PI3K/Akt↓; IL-6↓; MIP-1α, MIP-2, MCP-1↓; MDA, SOD↓ TNF-α, IL-6↓; ICAM-1↓; iNOS↓; M1↓; M2↑; pulmonary edema↓ TNF-α, IL-1β, STAT3, GATA3 ↓; T-bet, Foxp3 ↑	Sun et al. (2023) (Wu et al., 2024a) Li et al. (2022) (Su et al., 2023) (de Oliveira et al., 2019) (Jin et al., 2022) (Wang et al., 2022)
Flavonoids Alkaloids Terpenoids	Paeonol (PAE) Quercetin (QU) Pachypodol (Pac) Hesperidin (HPD) Luteolin (Lut) Baicalin Ligustrazine (LIG) Capsaicin (CAP) Tanshinone	PAE-CD-MOF QU-Nanogel MCQ/R Pac-lipo HPD/NPs CeLutNCs BA-LP LIG@OC-COF Fe-CAP TSIIA-NE	IL-1β, IL-6, IL-8, G-CSF↓ TNF-α, IL-1β, IL-6↓; MDA, SOD, CAT↑ TNF-α, IL-6, IL-1β↓; TLR4, NLRP3↓ TNF-α, IL-6, IL-1β, iNOS↓; TLR4-MyD88-NF-κB/MAPK↓; pulmonary edema, BALF protein content, spirometry↓ TNF-α, IL-1β, IL-6, IL-17↓; NO↓; lung dry weight/wet weight ratio of mice, BALF protein content↓ TNF-α, IL-1β, IL-6↓; NF-κB↓; iNOS↓; lung dry weight/wet weight ratio of mice↓ TNF-α, IL-1β↓; lung dry weight/wet weight ratio of mice↓; TLR4/JNK/ERK/NF-κB↓ TNF-α, IL-1β, IL-6, IL-8↓; Nrf2/NF-κB↓; MDA, SOD↓ TNF-α, IL-6↓; iNOS↓; NF-κB↓; lung dry weight/wet weight ratio of mice↓ TNF-α, IL-17↓; IL-10↑; SOD, MDA↓; lung dry weight/wet weight ratio of mice↓	Li et al. (2020) Chen et al. (2022b) (Zhang et al., 2024c) (Sun et al., 2024) (Jin et al., 2021) (Gu et al., 2024) (Long et al., 2020) (H et al., 2022) (Wang et al., 2024a) (El-Moslemany et al., 2022)
Glycosides Triterpenoids Coumarins	Ginsenoside Celastrol (Cel) Bergapten (Ber)	R@ZQC Cel-NPs Ber-lipo	TNF-α, IL-1β, IL-6, IL-17↓; MCP-1↓; NLRP3, GSDMD-N↓; ZBP1↓; NF-κB, AMPK, JAK-STAT↓ TNF-α, IL-1β, IL-6↓; NLRP3↓ TLR4/MyD88/NF-κB↓; TNF-α, IL-1β, IL-6, CD86, iNOS↓	Wu et al. (2024b) (Yao et al., 2024) Liao et al. (2025)

AMPK = AMP-activated protein kinase; BALF, bronchoalveolar lavage fluid; BSA, bovine serum albumin; CAT, catalase; CD, cyclodextrin; CS, chitosan; ERK, extracellular signal-regulated kinase; Foxp3 = forkhead box P3; GA, glycyrrhetinic acid; G-CSF, granulocyte colony-stimulating factor; GSDMD-N, gasdermin D N-terminal domain; HMSN, hollow mesoporous silica nanoparticles; HO, heme oxygenase; ICAM, intercellular adhesion molecule; iNOS, inducible nitric oxide synthase; JAK, janus kinase; JNK = c-Jun N-terminal kinase; LP, liposome; MAPK, mitogen-activated protein kinase; MCP, monocyte chemotactic protein; MDA, malondialdehyde; MIP, macrophage inflammatory protein; MOF, metal-organic framework; MPO, myeloperoxidase; NC, nanocomplex; NE, nanoemulsion; NF, nuclear factor; NLRP3 = NLR, family pyrin-domain-containing 3; NOX, NADPH, oxidase; NP, nanoparticle; PCGC, poly (citric acid-polyethylene glycol-curcumin); PI3K = phosphatidylinositol 3-kinase; PIP2 = phosphatidylinositol bisphosphate; PM, platelet membrane vesicle; SOD, superoxide dismutase; STAT, signal transducer and activator of transcription; T-bet = T-box transcription factor; TLR, toll-like receptor; TNF, tumor necrosis factor; TSIIA, tanshinone IIA; ZBP = Z-DNA-binding protein.

## 6 Challenges faced in the development of NP-NMs

Firstly, in terms of their preparation process, different types of NPs have different preparation methods. There is a lack of unified safety management, and unintentional release of NPs during production and processing is difficult to measure and track. This results in poor controllability and thus can be a great challenge for clinical conversion (Ramanathan, 2019). Secondly, in terms of drug delivery, the delivery of NPs faces multiple challenges, including shear, protein adsorption, and rapid clearance. These biological barriers become even more difficult to overcome in pathological states and thus limit the proportion of NPs that reach their targets (Mitchell et al., 2020; Blanco et al., 2015; Ensign et al., 2012). Furthermore, some NPs, such as liposomes, may be difficult to bind to the released drug in a controlled manner, which limits the controlled release effect (Cao et al., 2022). The mechanisms of the biocompatibility and toxicity of NPs are not yet fully understood (Mitchell et al., 2020; Ciganė et al., 2021). Unfortunately, although NPs have an important role in biomedicine, there is still very little information on studies of their long-term toxicity and analyses of their animal tolerance, which are necessary steps prior to human clinical trials (Mohammed-Sadhakathullah et al., 2023).

As a result of these challenges, we are therefore required to: 1) Aim to establish methods for the assessment of health risk during the preparation of NPs and prepare new exposure models that can track particles in the environment. 2) Continuously explore new options, such as intelligent nanoparticle design and responsive nanomaterials, so as to improve the targeting and controlled release of NPs. 3) Optimize the selection of nanomaterials–for example, by aiming to select materials that have been proved by existing studies to be highly biocompatible and have low toxicity–and continue to study the intrinsic mechanisms of the biocompatibility and toxicity of nanomaterials. 4) Develop a standardized manufacturing process to meet the requirements of drug regulatory agencies and more effectively facilitate the clinical conversion of NPs. Currently, a relatively large number of natural product nanomedicines have entered the clinical trial stage. Among them, curcumin-loaded nanoparticles represent the most extensively investigated formulations, having progressed through multiple phases of clinical evaluation. In addition, nanoparticle systems derived from other natural compounds have also been explored. For instance, clove extract-based nanoparticles have been evaluated for the treatment of dental caries, while quercetin-loaded polymeric nanoparticles have demonstrated promising activity in in vitro studies using oral cancer cell lines (Sanjai et al., 2024; Hassan et al., 2023). Nanoparticle albumin-bound paclitaxel (nab-paclitaxel), which is a nanoparticle derived from the natural product paclitaxel and bound to human serum protein. Owing to its favorable clinical efficacy and safety profile, nanoparticle albumin-bound paclitaxel (nab-paclitaxel) has been approved by the U.S. Food and Drug Administration (FDA) for the treatment of metastatic breast cancer, non-small cell lung cancer (NSCLC), and other malignancies. In addition, the combination of nab-paclitaxel with gemcitabine has become a standard first-line therapy for metastatic pancreatic cancer (MPC), as demonstrated by the positive outcomes of the global phase III MPACT trial (Kim, 2017). Moreover, emerging clinical evidence supports the potential utility of nab-paclitaxel, either as monotherapy or in combination with other agents, in the management of metastatic esophageal, gastric, colorectal, and biliary tract cancers (Hassan et al., 2023).

## 7 Conclusion and perspective

Natural products are widely used in biomedicine, genetic engineering, and other fields because of their natural biocompatibility, biodegradability, and other intrinsic properties, but their poor bioavailability limits their exploitation. By combining natural products with nanomaterials, new strategies are provided for natural products to utilize their unique advantages, improve their bioavailability, expand the scope of targeted therapies, and take advantage of controlled release. These provide new ideas for the treatment of ALI. In addition, compared with traditional treatment methods, the initial development and preparation costs of NP-NMs are relatively high. However, from the perspective of long-term treatment efficacy, NP-NMs, with their excellent targeting properties, can reduce the number of doses required and shorten the treatment cycle, thereby lowering costs. This is particularly advantageous in the management of chronic diseases, where they offer significant economic benefits.

However, promoting the translation of NP-NMs to clinical practice is an arduous process that requires a large number of basic experiments and clinical trials to advance the widespread clinical application of NP-NMs. We will vigorously promote standardized preparation processes for NP-NMs, long-term toxicology research, and research on multi-mechanism coordinated release control combined with AI development (Joyce et al., 2024). With the continuous development of nanotechnology and the exploration of the mechanisms of natural products, there is no doubt that NP-NMs hold promise and will become important drugs for the treatment of ALI and other diseases in the future.
